# Forward modelling low-spectral-resolution Cassini/CIRS observations of Titan

**DOI:** 10.1007/s10686-024-09934-y

**Published:** 2024-03-21

**Authors:** Lucy Wright, Nicholas A. Teanby, Patrick G. J. Irwin, Conor A. Nixon

**Affiliations:** 1 School of Earth Sciences, University of Bristol, Bristol, UK; 2 Atmospheric, Oceanic, and Planetary Physics, Department of Physics, University of Oxford, Oxford, UK; 3 Planetary Systems Laboratory, NASA Goddard Space Flight Centre, Greenbelt, MD 20771 USA

**Keywords:** Radiative transfer(1335), Infrared spectroscopy(2285), Titan(2186)

## Abstract

The Composite InfraRed Spectrometer (CIRS) instrument onboard the Cassini spacecraft performed 8.4 million spectral observations of Titan at resolutions between 0.5–15.5 cm^-1^. More than 3 million of these were acquired at a low spectral resolution (SR) (13.5–15.5 cm^-1^), which have excellent spatial and temporal coverage in addition to the highest spatial resolution and lowest noise per spectrum of any of the CIRS observations. Despite this, the CIRS low-SR dataset is currently underused for atmospheric composition analysis, as spectral features are often blended and subtle compared to those in higher SR observations. The vast size of the dataset also poses a challenge as an efficient forward model is required to fully exploit these observations. Here, we show that the CIRS FP3/4 nadir low-SR observations of Titan can be accurately forward modelled using a computationally efficient correlated-$\varvec{k}$ method. We quantify wavenumber-dependent forward modelling errors, with mean 0.723 nW cm$^{\varvec{-2}}\,$sr^-1^/cm^-1^ (FP3: 600–890 cm^-1^) and 0.248 nW cm$^{\varvec{-2}}\,$sr$^{\varvec{-1}}\,$/ cm^-1^ (FP4: 1240–1360 cm^-1^), that can be used to improve the rigour of future retrievals. Alternatively, in cases where more accuracy is required, we show observations can be forward modelled using an optimised line-by-line method, significantly reducing computation time.

## Introduction

The Cassini spacecraft explored Saturn and its moons for 13 years (2004–2017). During that time, Cassini performed 127 close flybys of Saturn’s largest moon, Titan, observing it for almost half of a Titan year, which is approximately 29.5 Earth years. Titan is the only moon in our solar system with a substantial atmosphere, and is host to many hydrocarbon (C_x_H_y_) and nitrile (C_x_H_y_N_z_) species produced by complex photochemistry in Titan’s upper atmosphere [30]. Many species in Titan’s atmosphere are infrared-active, so observations in the far- and mid-infrared are important for characterising Titan’s atmospheric chemistry and dynamics [5, 23, 25].

The Composite InfraRed Spectrometer (CIRS) [8, 14, 15, 17] onboard the Cassini spacecraft acquired 8.4 million spectral observations of Titan. CIRS comprised two interferometer spectrometers, sensitive to the far- to mid-infrared spectral region (10–1500 cm^-1^) across three focal planes: FP1 (10–600 cm^-1^), FP3 (600–1100 cm^-1^), and FP4 (1100–1500 cm^-1^). CIRS had an adjustable spectral resolution (SR), typically observing in one of three SR modes: high SR (full width at half maximum, FWHM $\sim$ 0.5 cm^-1^), medium SR (FWHM $\sim$ 2.5 cm^-1^) and low SR (FWHM $\sim$ 14.5 cm^-1^).

**Fig. 1:**
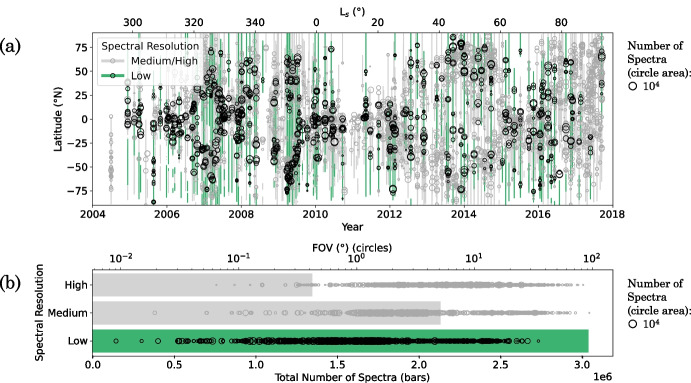
Mission coverage of all CIRS nadir observations of Titan, with low-spectral resolution (SR) observations highlighted in green. a: Latitude coverage (bars) and mean latitude (circles) of each observation at the time of observation. b: Total number of spectra (shaded regions) acquired by CIRS at high (FWHM $\sim$ 0.5 cm^-1^), medium (FWHM $\sim$ 2.5 cm^-1^), and low (FWHM $\sim$ 14.5 cm^-1^) SR, and field of view (FOV) (circles) of each observation. Circle area is proportional to the number of spectra in that observation. A circle area corresponding to $10^4$ spectra is shown for scale. Smaller FOV corresponds to higher spatial resolution. A large proportion of CIRS spectra have a low SR, and low-SR observations have good spatial and temporal coverage throughout the mission (2004–2017). Low-SR observations typically have a smaller FOV size and hence a higher spatial resolution

**Fig. 2:**
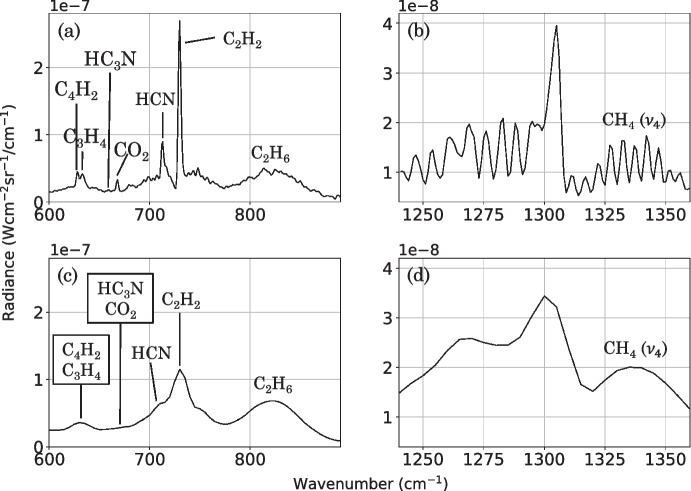
Example CIRS FP3/4 measured Titan spectrum at a medium SR (FWHM = 2.5 cm^-1^) (a, b) and a low SR (FWHM = 14.5 cm^-1^) (c, d). Emission peaks of key gases in Titan’s atmosphere are labelled. A typical noise on an individual spectrum observed at a low-SR ($\sim 2.5$ nW cm$^{-3}\,$sr$^{-1}\,$/ cm^-1^ for FP3 and $\sim 0.5$ nW cm$^{-3}\,$sr$^{-1}\,$/ cm^-1^ for FP4) is less than on an individual spectrum observed at a medium-SR ($\sim 8$ nW cm$^{-3}\,$sr$^{-1}\,$/ cm^-1^ for FP3 and $\sim 1.5$ nW cm$^{-3}\,$sr$^{-1}\,$/ cm^-1^ for FP4). In the low-SR measured spectra, HCN, C_2_H_2_, C_2_H_6_, and CH_4_ peaks are distinct, but C_4_H_2_ and C_3_H_4_ peaks are blended. HC_3_N and CO_2_ peaks are also blended

The low-SR observations have excellent spatial resolution, noise properties, and coverage, but they are the least used type of CIRS observation. Low-SR observations require shorter acquisition times, so could be performed when the spacecraft was in closer proximity to Titan. This had two important consequences: (i) more spectra could be acquired within a given time interval, leading to a higher signal-to-noise ratio when co-adding spectra; and (ii) observations could be taken closer to Titan’s surface, resulting in the highest horizontal spatial resolution among all CIRS observations (Fig. 1). As a result, these observations may reveal atmospheric changes over smaller horizontal distances that might not be captured in higher SR observations. In addition, low-SR observations typically yield the lowest noise per spectrum among all CIRS observations (e.g., Fig. 2), and, with more than 3 million low-SR CIRS observations of Titan acquired, they represent the most numerous of any CIRS observation type (Fig. 1). These observations offer excellent latitudinal and temporal coverage throughout the full Cassini mission (Fig. 1), valuable for monitoring seasonal change in Titan’s atmosphere.

Low-SR observations were acquired in FP4 primarily for temperature mapping [8], with FP1 and FP3 observations taken at the same time. CIRS FP3/4 low-SR nadir observations have a FWHM between 12.6–14.7 cm^-1^, and an average FWHM of 14.25 cm^-1^. Low-SR limb observations have been used extensively for temperature analysis [1, 24, 28, 29], but low-SR nadir observations are underused for atmospheric composition analysis. This is because spectral features are subtle compared to those in higher SR nadir observations, and spectral features can often become blended. Consequently, modelling this dataset requires greater attention.

Efficient and accurate forward modelling of these spectra is essential to make use of this large dataset. We explore a correlated-*k* (c-*k*) [16] approach and the optimisation of a line-by-line (LBL) method. The c-*k* method is used extensively (e.g., Teanby et al. [22], Cottini et al. [6], Sharkey et al. [20], Sylvestre et al. [21]) to forward model Cassini/CIRS spectral observations of Titan acquired with a FWHM less than 3 cm^-1^. However, the c-*k* method may be less accurate at modelling the blended peaks in low-SR CIRS observations. We evaluate the accuracy of both c-*k* and LBL methods to forward model low-SR CIRS FP3/4 nadir Titan observations, with a consideration for computational efficiency. These observations are likely to remain the most numerous and highest spatial resolution mid-IR data we have of Titan for the next two decades at least. So this is a critical step to ensure these data can be fully exploited.

**Fig. 3:**
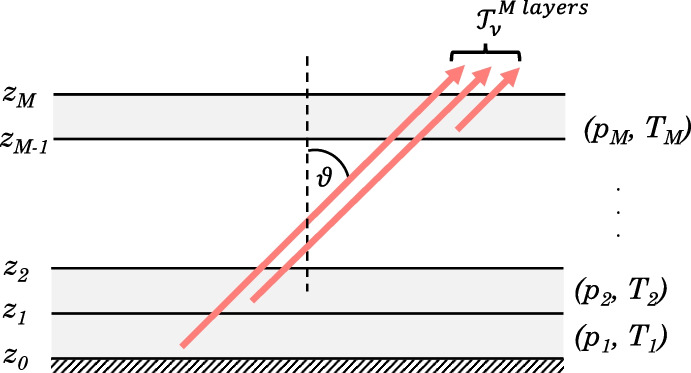
An atmosphere can be modelled as *M* homogeneous layers, each of a constant pressure, $p_i$, and temperature, $T_j$. Observing along some path through an atmosphere, at zenith angle $\theta$, the transmission at the top of the atmosphere, $\mathcal {T}_{\nu }^{M\,layers}$, can be found by summing contributions from each homogeneous layer. Emission of radiation, along the viewing path, from each atmospheric layer is represented by red arrows

## Line-by-line forward model

**Fig. 4:**
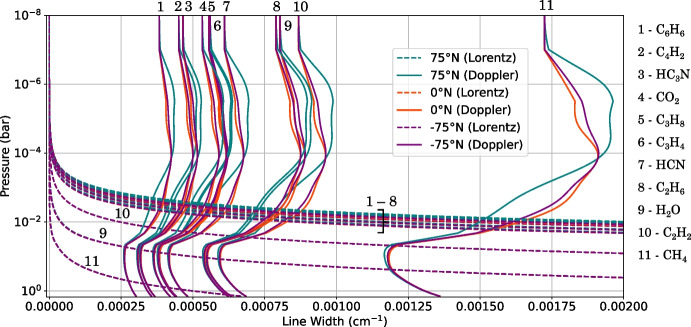
Example Lorentz- and Doppler-broadened spectral line widths for some spectrally active gases in Titan’s atmosphere. The Lorentz line width (dashed lines) is due to collisions between molecules, and is dependent on atmospheric temperature and pressure. The Doppler line width (solid lines) is due to the relative velocity of a molecule with respect to the observer, and is dependent on atmospheric temperature. Typical values based on line data from HITRAN and GEISA. Line widths are shown at three latitude end members: 75^∘^N (blue), 0^∘^N (orange), and -75^∘^N (purple) in Titan northern mid-winter (year 2005). Representative temperature profiles are taken from Teanby et al. [25] (Supplementary Material S3). The narrowest HWHM line width is approximately $4 \times 10^{-4}$ cm^-1^ (C_6_H_6_). Hence, in a LBL forward model, a grid spacing of *gs* = $2\sqrt{2\ln 2} (4 \times 10^{-4}$ cm$^{-1}) \approx 9.4 \times 10^{-4}$ cm^-1^ is required to Nyquist sample the minimum line width

To model the emission from Titan’s atmosphere, we use the Non-linear Optimal Estimator for MultivariatE Spectral AnalySIS (NEMESIS) [12] with Titan’s atmospheric temperature and composition based on Teanby et al. [25]. To determine how accurate the resulting forward-modelled spectrum is, it is important to consider the key elements which contribute to the radiative transfer calculation. An inhomogeneous atmosphere can be modelled as *M* homogeneous layers. NEMESIS uses a spherical layering scheme [12], but here we illustrate this problem using a plane-parallel case for simplicity (Fig. 3). The transmission of monochromatic radiation, of wavenumber $$\nu _0$$, through an atmosphere can be calculated by summing over all absorption lines, $$i = 1, ..., N$$, in all layers, $$j = 1, ..., M$$, for all gases, $$l= 1, ..., L$$. We use $$M = $$ 99 layers to model Titan’s atmosphere. Transmission along a path of zenith angle $$\theta $$ (Fig. 3) is given by

1 $$\begin{aligned} \mathcal {T}^{M\,layers,\,L\,gases}(\nu _0) \!=\! \exp \! \left[ \!-\frac{1}{cos\theta } \!\sum ^M_{j=1} n(z_j) \!\left( \sum _{l=1}^L q_l(z_j) \!\sum _{i=1}^{N} k_{ijl}\left( \nu _0, p_j, T_j\right) \!\right) \! \Delta z_j \!\right] \end{aligned}$$

(e.g., Goody and Yung [9], Irwin [13]), where $n(z_j)$ is the number density of molecules (molecules/m^3^), $q_l(z_j)$ is the mole fraction of gas *l*, and $z_j$ is the altitude, and $\Delta z_j$ is the thickness, of the $j^{th}$ atmospheric layer which has pressure $p_j$ and temperature $T_j$. The absorption coefficient, $k_{ijl}$, of each spectral line at wavenumber $\nu _0$ must be calculated using the line shape and the line strength, given by line databases. We assume the atmosphere is non-scattering in the CIRS range < 1400 cm^-1^ ($\gtrsim$ 7 $\mu$m) as wavelengths ($\sim$ 25–2.5 $\mu$m) are generally larger than Titan’s cloud and haze particle size (0.0013–3.35 $\mu$m, Toon et al. [26], Barth [2]). Computing an emission spectrum line-by-line (LBL), where the contribution from individual spectral lines is considered, is the most accurate method of forward modelling. In a LBL forward model, the absorption, $k_{ijl}(\nu )$, of each individual spectral line is calculated for each atmospheric pressure and temperature ($p_j$, $T_j$). We calculate absorptions using line strengths, widths and broadenings provided by line databases HITRAN [10] and GEISA [7]. Absorption coefficients vary rapidly with wavenumber so, when summing absorptions line-by-line (LBL), a fine underlying grid spacing, *gs*, smaller than the width of the narrowest line in the spectral region of interest, is required to resolve every spectral line in that region. The spectral grid spacing required is planet dependent as spectral line widths are temperature and pressure-dependent due to multiple broadening processes: most predominately Lorentz and Doppler broadening (e.g., Fig. 4). Lorentz broadening is due to collisions between molecules. Higher atmospheric pressure or temperature increases the frequency of collisions between molecules. More frequent collisions shorten the radiation absorption/emission time, $\Delta t$, so, by the Uncertainty Principle [4]: $\Delta E \Delta t \gtrsim \frac{h}{4\pi }$, where *h* is the Planck constant, the uncertainty in the wavenumber value, $\Delta \nu$ ($\propto \Delta E$, the uncertainty in energy) increases. Therefore, the spread in wavenumbers, $\Delta \nu$, increases and the line is broadened. Doppler broadening is due to the direction of molecule movement relative to the observer. Molecules moving towards the observer will appear to emit higher wavenumber radiation, and molecules moving away will appear to emit lower wavenumber radiation, due to the Doppler effect. Molecules move with velocities distributed with a Maxwell-Boltzmann distribution, dependent on the molecular mass and temperature of the gas. The Doppler line width is therefore dependent on temperature, but not pressure, and so follows the form of the atmospheric temperature profile (Fig. 4). At higher pressures, Lorentz broadening dominates.

### Reference line-by-line spectrum

The narrowest spectral line emitted by any spectrally active gas in Titan’s atmosphere in the CIRS FP3/4 spectral range has HWHM $$= 4 \times 10^{-4}$$ cm^-1^ (Fig. 4), and is controlled by the line width due to Doppler broadening. It is critical that the underlying grid spacing, *gs*, of the LBL spectrum is less than the width of the narrowest spectral line such that all lines are resolved. To minimally resolve a signal, or to “Nyquist” sample a signal, the sampling frequency must be at least twice the maximum frequency of the signal [19]. The Doppler line shape is a Gaussian, and has HWHM = $$\sqrt{2\ln 2} \gamma _D$$. Approximating the central peak of the Gaussian to half a sine wave, the maximum spacing required to achieve Nyquist sampling is equal to the FWHM. Hence, the maximum grid spacing required to resolve every spectral line in the FP3/4 Titan spectrum can be determined from the minimum line width: $$gs_{max} = $$ FWHM $$=2[$$HWHM$$]= 2[\sqrt{2\ln 2} \gamma _D^{min}] = 2\sqrt{2\ln 2}(4 \times 10^{-4}$$ cm$$^{-1}) \approx 9.4 \times 10^{-4}$$ cm^-1^

**Fig. 5:**
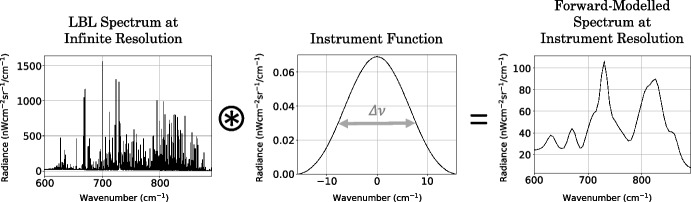
Convolution of an example LBL infinite resolution synthetic spectrum with an instrument function to produce a finite resolution spectrum. The spectrum emitted by an atmosphere has an infinite resolution. In observing the emission, the spectrum is smoothed by the finite-resolution viewing instrument. The resulting observed spectrum can be calculated by convolution of the infinite resolution spectrum with the viewing instrument’s apodisation function. The Hamming function is shown here as an example instrument function

We compute a high-accuracy FP3/4 synthetic Titan spectrum by performing a LBL forward model with an underlying spectral grid spacing of $gs = 2 \times 10^{-4}$ cm^-1^, to oversample $gs_{max}$ by at least a factor of 4. The resulting LBL computed spectrum is effectively the infinite resolution emission spectrum expected from our prescribed *a priori* atmospheric state. In reality, the viewing instrument will have some finite resolution and the observed spectrum is consequently smoothed (Fig. 5). To model an observed spectrum, the infinite resolution spectrum is convolved with the apodisation function associated with the viewing instrument. We refer to the synthetic spectrum produced with a grid spacing of $gs = 2 \times 10^{-4}$ cm^-1^ as the *gold-standard* synthetic spectrum, and use it as a reference to assess the accuracy of other forward models.

### Line-by-line modelling at reduced resolution

**Fig. 6:**
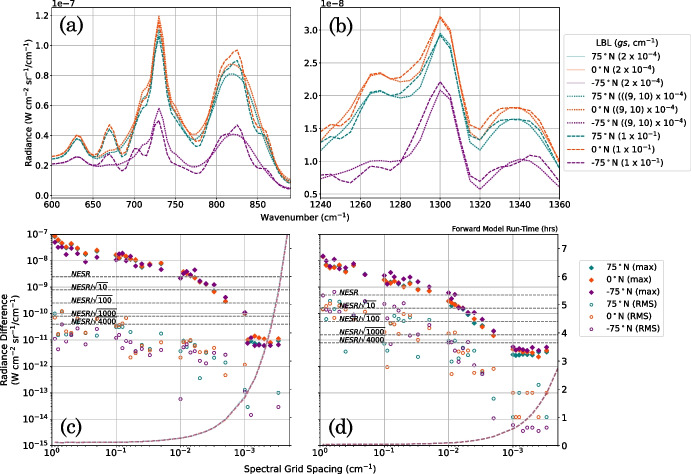
Comparison of synthetic Titan spectra produced with a line-by-line (LBL) forward model at varied underlying spectral grid spacing. a, b: Synthetic CIRS FP3 (a) and FP4 (b) Titan spectra computed with a LBL forward model are shown at three grid spacing end members – fine ($gs = 2 \times 10^{-4}$ cm^-1^, the *gold-standard* grid spacing which Nyquist samples the narrowest spectral line, solid line), coarse ($gs = 1 \times 10^{-1}$ cm^-1^, dashed line) and an optimal ($gs = 9 \times 10^{-4}$ cm^-1^ for FP3 and $gs = 10 \times 10^{-4}$ cm^-1^ for FP4, dotted line) grid spacing. c, d: FP3 (c) and FP4 (d) synthetic spectra computed with each underlying grid spacing are subtracted from the *gold-standard* synthetic spectrum. The maximum (max, diamonds) and root-mean-squared (RMS, circles) absolute radiance difference between the spectra are shown at three latitude end-members: 75^∘^N (blue), 0^∘^N (orange), and -75^∘^N (purple) in Titan northern mid-winter (year 2005). A typical run-time to compute one synthetic Titan CIRS FP3/4 spectrum at each coarse grid spacing is also shown (coloured dashed lines). Representative NESR levels (2.5 nW cm$^{-3}\,$sr$^{-1}\,$/ cm^-1^ for FP3, 0.5 nW cm$^{-3}\,$sr$^{-1}\,$/ cm^-1^ for FP4, taken from Flasar et al. [8]) are labelled (grey dashed lines), including the limiting noise NESR/$\sqrt{4000}$. CIRS FP3/4 Titan nadir spectra can be forward modelled within the limiting noise level using a grid spacing less than the width of the narrowest spectral line in the FP3/4 region

A large computation cost of calculating a LBL spectrum is due to the very fine grid spacing required to resolve all spectral lines. We explore the effect of relaxing this constraint on forward modelling accuracy by assessing the accuracy of the LBL forward model at different underlying spectral grid spacings. We compute synthetic FP3/4 Titan spectra using a LBL forward model with 35 spectral grid spacing values in the range $(2 \times 10^{-4})$–$(9 \times 10^{-1})$ cm^-1^ and compare each to the *gold-standard* synthetic spectrum. The forward model is performed with *a priori* profiles of temperature and gas volume mixing ratio (VMR) at three example latitude end members (75^∘^N, 0^∘^N, and -75^∘^N) at one example year (2005): when Titan’s northern hemisphere was in mid-winter. We use these end members as representatives of northern (mid-winter), equatorial, and southern (early summer) atmospheric states, respectively. Temperature profiles are taken from Teanby et al. [25] (Supplementary Material S3) and include constraints from CIRS FP1 nadir observations [21], FP4 nadir observations [24], and FP4 low-resolution limb observations [24]. Example end members of LBL forward-modelled synthetic spectra are shown in Fig. 6. Figure 6 shows the mean and maximum radiance difference between each spectra with respect to the *gold-standard* spectrum, and also a typical computation run-time of the LBL forward model at each spectral grid spacing. For finer spectral grid spacing, the computation run-time of the LBL forward model rapidly increases (Fig. 6).

The accuracy required for the LBL forward model is determined by the absolute noise level. For a spectral radiance measurement, the noise-equivalent spectral radiance (NESR) is the radiance incident on the detector for which the signal-to-noise ratio, S/N = 1 [11]. The NESR encapsulates all sources of noise in the detection of an infrared signal. The noise in a single CIRS spectrum is given by the NESR which has a wavenumber depenence but is approximately equal to 2.5 nW cm$^{-2}\,$sr$^{-1}\,$/ cm^-1^ for FP3 and 0.5 nW cm$^{-2}\,$sr$^{-1}\,$/ cm^-1^ for FP4 for the low-SR observations, ignoring the detector edges [8]. To increase S/N, spectra are typically averaged together based on spatial bins. For an average of $N_{av}$ spectra, the noise will be NESR$/\sqrt{N_{av}}$. However, each spectrum in an observation sequence has a background deep space spectrum subtracted to remove instrumental self-emission [14]. Here, background emission is removed from measured spectra by performing a DS-4000 calibration – where the background spectrum for each observation sequence is constructed from the 4000 nearest deep space spectra – as used by Teanby et al. [25]. Consequently, the limiting noise on our calibrated spectra, excluding modelling errors, is NESR$/\sqrt{4000}$, no matter how many spectra from an observation sequence are averaged together.

Figure 6 can be used to determine the optimal forward-model grid spacing for a given observation noise level. The maximum spectral grid spacing permitted to accurately LBL forward model Titan CIRS FP3/4 nadir spectra within the limiting noise level (NESR/$\sqrt{4000}$) is determined to be $gs = 9 \times 10^{-4}$ cm^-1^ for FP3 and $gs = 10 \times 10^{-4}$ cm^-1^ for FP4 (Fig. 6). This is consistent with the above estimated maximum grid spacing required to resolve every spectral line ($gs_{max}\approx 9.4 \times 10^{-4}$ cm^-1^). Computation of one synthetic spectrum at this grid spacing typically requires 115 minutes for FP3 and 36 minutes for FP4 (Fig. 6): a significantly reduced run-time compared to using, for example, the *gold-standard* grid spacing ($gs = 2 \times 10^{-4}$ cm^-1^) which typically requires 8 hours for FP3 and 3 hours for FP4. The optimal run parameters required to achieve some example noise levels are given in Table 1. A larger grid spacing can be used to achieve noise levels greater than the limiting noise level, at a significantly reduced computation cost (Fig. 6, Table 1). The maximum radiance difference between the synthetic spectrum produced using the *gold-standard* grid spacing ($gs = 2 \times 10^{-4}$ cm^-1^) and coarser grid spacing values converges at approximately $1 \times 10^{-2}$ nW cm$^{-2}\,$sr$^{-1}\,$/ cm^-1^ for FP3 and $5 \times 10^{-3}$ nW cm$^{-3}\,$sr$^{-1}\,$/ cm^-1^ for FP4 (Fig. 6), which is at least three orders of magnitude smaller than the radiance of a CIRS FP3/4 Titan spectrum, which is typically 10^1^–10^2^ nW cm$^{-2}\,$sr$^{-1}\,$/ cm^-1^. The very small remaining difference is due to numerical precision of the current NEMESIS code. We note that because of the low SR, a pre-convolution spectrum equal to the width of the CIRS instrument function is required for each wavenumber. This is $\sim 31.4$ cm^-1^ for a FWHM of 14.25 cm^-1^. Therefore, a grid spacing of $2 \times 10^{-4}$ cm^-1^ requires $\sim (31.4$ cm$^{-1})/(2 \times 10^{-4}$ cm$^{-1}) \sim 160,000$ points and is the smallest spacing that can be used without requiring unfeasibly large array sizes. We also note that the convergence of grid spacing values begins at approximately $gs = 9 \times 10^{-4}$ for FP3 and $gs = 10 \times 10^{-4}$ cm^-1^ for FP4 (Fig. 6). This is, as expected, approximately equal to the maximum grid spacing required to resolve every spectral line, $gs_{max} \approx 9.4 \times 10^{-4}$ cm^-1^, which was determined above using the minimum spectral line width and Nyquist sampling.

**Table 1:** Maximum underlying spectral grid spacing permitted for a line-by-line (LBL) forward model to produce spectra accurate to within some example noise levels

	75^∘^N		0^∘^N		-75^∘^N	
	Grid Spacing	Run-time	Grid Spacing	Run-time	Grid Spacing	Run-time
	(cm^-1^)	(minutes)	(cm^-1^)	(minutes)	(cm^-1^)	(minutes)
*NESR*	0.006, 0.01	22, 6	0.006, 0.01	23, 6	0.005, 0.007	26, 7
$\frac{NESR}{\sqrt{10}}$	0.003, 0.006	39, 8	0.003, 0.005	38, 9	0.002, 0.005	56, 9
$\frac{NESR}{\sqrt{100}}$	0.001, 0.003	105, 13	0.001, 0.003	102, 14	0.001, 0.002	102, 20
$\frac{NESR}{\sqrt{1000}}$	0.0009, 0.002	116, 19	0.0009, 0.002	114, 19	0.0009, 0.001	114, 36
$\frac{NESR}{\sqrt{4000}}$	0.0009, 0.001	116, 36	0.0009, 0.001	114, 36	0.0009, 0.001	114, 36

A typical computation run-time to perform one forward model at each grid spacing is also given. The grid spacing is given to one significant figure and run-times to the nearest minute. Approximate noise equivalent spectral radiance (NESR) levels are taken from Flasar et al. [8]). For comparision, the computation run-time for a LBL forward model with an underlying grid spacing of $gs = 2 \times 10^{-4}$ cm^-1^ is typically 8 hours for FP3 and 3 hours for FP4

**Fig. 7:**
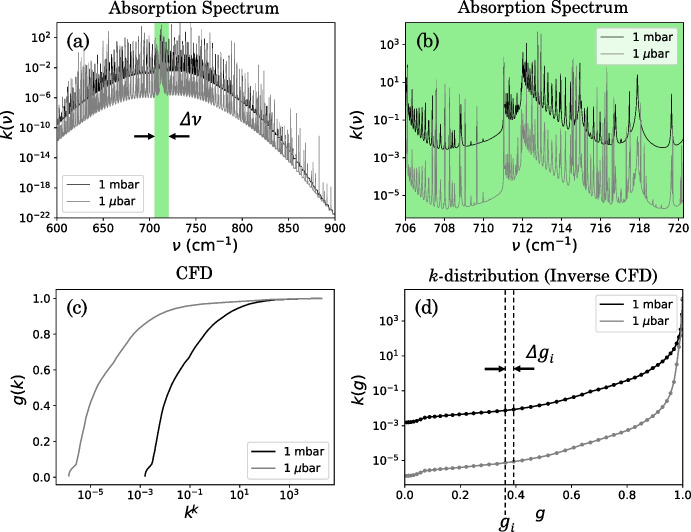
An example HCN absorption spectrum and *k*-distribution at two example altitudes in Titan’s atmosphere. a: A HCN absorption spectrum in the CIRS FP3 spectral range. b: The same spectrum but zoomed to a smaller spectral interval, with width equal to the average FWHM of CIRS low-spectral resolution (SR) observations ($\Delta \nu =$ 14.25 cm^-1^). This region is highlighted in green in a. c: Cumulative frequency distribution (CFD) of the absorption spectrum. d: The CFD is inverted to obtain a *k*-distribution. The *k*-distribution is sampled at *NG* *g*-ordinates following a Gaussian quadrature scheme. Ordinates are shown as circular points in d, where $NG =$ 50 in this example. Each is shown at two example altitudes in Titan’s atmosphere: 1 mbar (150 K, black line) and 1 $\mu$bar (170 K, grey line). The absorption spectrum (a) varies rapidly with wavenumber, whereas the *k*-distribution (d) is a smooth function in *g*-space which allows for coarser sampling and hence a computationally quicker forward model

## Correlated-*k* Forward Model

We have shown that Cassini/CIRS FP3/4 nadir spectral observations of Titan can be forward-modelled using a LBL approach optimised for computational efficiency. We now explore a second approach to forward modelling – the correlated-*k* (c-*k*) method [9, 16] – to further improve computational efficiency.

Absorption spectra vary rapidly with wavenumber (e.g., Fig. 7a). As a result, summing absorption coefficients LBL can make forward modelling very computationally intensive. In general, we don’t require an infinite resolution spectrum as observations are at a much lower resolution. The standard c-*k* approach to forward modelling considers a finite spectral interval and reproduces the instrument function with a post convolution. The c-*k* method is an approximation which relies on the assumption that the strongest (weakest) absorption lines in one atmospheric layer are well-correlated with the strongest (weakest) lines in the adjacent layers [16]. Under this assumption, it does not matter where in the spectrum each absorption coefficient occurs, so absorption lines can be reordered, by size. This reordering results in a smooth function. This allows absorptions to be pre-tabulated on a fine grid, but then re-ordered and tabulated on a much coarser grid for subsequent analysis. Such tables are time consuming to produce as they are generated using the LBL method, but critically only need to be generated once so are well suited to rapid forward modelling of many spectra. This makes the c-*k* method of forward modelling much faster than the LBL method.

Consider a nadir observation of a homogeneous (single-layer) atmosphere. Observing at spectral resolution $\Delta \nu$, the mean transmission of radiation in the spectral interval $\nu _0 \rightarrow \nu _0 + \Delta \nu$ observed at the top of the atmosphere can be calculated by summing over all spectral lines in that range (e.g., Goody and Yung [9]):

2 $$\begin{aligned} \overline{\mathcal {T}}^{single\,layer}(n) = \frac{1}{\Delta \nu } \int ^{\nu _0 + \Delta \nu }_{\nu _0} e^{-n k(\nu )} d\nu , \end{aligned}$$

where $k(\nu )$ is the total absorption coefficient from all spectral lines at wavenumber $\nu$, and *n* is the number density of molecules along the path. Absorption coefficients can be summed in any order, not affecting the total transmission. It doesn’t matter where in the spectral interval $\nu _0 \rightarrow \nu _0 + \Delta \nu$ an absorption coefficient has some value in the range $k \rightarrow k + dk$, only what fraction of the spectral interval absorption coefficients in that range occupy. So the transmission function (Equation 2) can equally be written as

3 $$\begin{aligned} \overline{\mathcal {T}}^{single\,layer}(n) = \int ^{\infty }_{0} f(k) e^{-n k} dk, \end{aligned}$$

where *f*(*k*) is the frequency distribution of absorption coefficients [16]. Now, instead of summing the absorption coefficients in wavenumber space, the percentage of frequency space occupied by absorption coefficients $k \rightarrow k+dk$ is computed. It is numerically easier to write this distribution of absorption coefficients as a cumulative frequency distribution (CFD), which is a smoothly varying monotonic function (Fig. 7c). We can define a function *g*(*k*) as the cumulative sum of *f*(*k*),

4 $$\begin{aligned} g(k) = \int ^{k}_0 f(k) dk. \end{aligned}$$

Hence,

5 $$\begin{aligned} dg= f(k)dk, \end{aligned}$$

such that (3) may be written as

6 $$\begin{aligned} \overline{\mathcal {T}}^{single\,layer}(n) = \int ^{1}_{0} e^{-n k(g)}dg, \end{aligned}$$

[16], where *k*(*g*) is the inverted CFD and known as the *k*-distribution (Fig. 7d). Comparing (2) to (6), we can see that variables $\nu$ and *g* are interchangeable when calculating the transmission. Since *g*(*k*) is a smoothly varying function, this can be reliably approximated to

7 $$\begin{aligned} \overline{\mathcal {T}}^{single\,layer}(n) \approx \sum ^{NG}_{i=1} e^{-n k_i(g)} \Delta g_i, \end{aligned}$$

where the *k*-distribution, *k*(*g*), is sampled at ordinates $i = 1, ..., NG$ in *g*-space. The *k*-distribution is essentially the absorption spectrum re-grouped and re-ordered by absorption coefficient strength. The *k*-distribution has value $k_i$ and weighting $\Delta g_i$ at the $i^{th}$ *g*-ordinate, where $\Delta g_i$ is the spacing between *g*-ordinates *i* and $i+1$. Unlike $k(\nu )$ (Fig. 7a), the *k*-distribution, *k*(*g*) (Fig. 7d), is a smoothly varying function. We can therefore sum over *k*(*g*) with a much coarser grid in *g*-space than in wavenumber space, whilst including contributions from all spectral lines. A *k*-distribution function for each gas can be pre-tabulated by calculating a high resolution LBL absolute *k* spectrum, reordering, and fitting with *NG* *g*-ordinates as a function of pressure, temperature, and wavenumber. These *k*-tables are time consuming to produce but once done vastly speed up the forward modelling.

A real atmosphere is not homogeneous but can be accurately modelled as *M* homogeneous layers (Fig. 3), each with some constant pressure and temperature $(p_j, T_j)$. Consider the plane parallel case for simplicity. The mean total transmission of radiation in a spectral region $\nu _0 \rightarrow \nu _0 + \Delta \nu$ at the top of an atmosphere, along a path through homogeneous layers $j = 1, ..., M$ is the product of mean transmissions from all layers. So, by equation (2),

8 $$\begin{aligned} \overline{\mathcal {T}}^{M\,layers} = \frac{1}{\Delta \nu }\int ^{\nu _0 + \Delta \nu }_{\nu _0} \exp \left[ -\sum _{j=1}^{M}n_jk_{j}(\nu )\right] d\nu , \end{aligned}$$

where $k_j$ is the total absorption coefficient of the $j^{th}$ layer. Variables $\nu$ and *g* are again interchangeable, assuming that absorption strengths within each spectral interval are well-correlated between atmospheric layers [16]. Interchanging $\nu$ and *g* gives

9 $$\begin{aligned} \overline{\mathcal {T}}^{M\,layers} = \int ^{1}_{0} \exp \left[ -\sum _{j=1}^{M}n_{j} k_{j}(g)\right] dg, \end{aligned}$$

which can be approximated to

10 $$\begin{aligned} \overline{\mathcal {T}}^{M\,layers} = \sum ^{NG}_{i=1} \exp \left[ -\sum _{j=1}^{M}n_{j} k_{ij}(g)\right] \Delta g_i. \end{aligned}$$

As with the single-layer case, the mean transmission from *M* layers can be determined from pre-tabulated *k*-distributions, *k*(*g*), sampled at ordinates $i = 1, ..., NG$ in *g*-space. If the correlated-*k* assumptions hold, then the difference in accuracy between the c-*k* method and LBL method should be minimal and only due to the interpolation of the smooth function *k*(*g*). In general, performing the sum in equation (7) with a finer *g*-space interval, $\Delta g_i$, and with a larger number of *g*-ordinates, *NG*, gives a more accurate representation of the *k*-distribution. This makes for a more accurate forward model, but at an increased computation cost. Inaccuracies can arise when the *k*-distribution is sampled at an insufficient number of *g*-ordinates to resolve the rapid increase in absorption coefficient, *k*(*g*), that typically occurs at the edges of a *k*-distribution (e.g. Fig. 7d), particularly for layers with sparse and narrow spectral lines. Therefore a Gauss-Lobatto sampling scheme [18] is typically used to decrease grid spacing near ends of the domain.

### Pre-tabulating the *k*-distribution: *k*-tables

Prior to performing a c-*k* forward model, the *k*-distribution function, *k*(*g*), is calculated and tabulated into ‘*k*-tables’ for each absorbing gas in the atmosphere of interest. First, for each spectral interval, $\Delta \nu$, an absorption spectrum, $k(\nu )$, is calculated at many pressures and temperatures in the atmosphere using line strengths, widths and broadenings provided by line databases HITRAN [10] and GEISA [7]. The width of the spectral interval $\Delta \nu$ is chosen based on the resolution of the viewing instrument. (e.g., Fig. 7). Here, we use a spectral interval of width equal to the average FWHM of the CIRS instrument in the low SR mode: $\Delta \nu = 14.25$ cm^-1^. Using the absorption spectrum, a CFD, *g*(*k*), (e.g., Fig. 7c) is found by computing the fraction of wavenumbers, or tiny wavenumber intervals, $\delta \nu$, within the wavenumber region $\Delta \nu$, with absorption coefficient strength less than some limiting value $k_L$:

11 $$\begin{aligned} g(k_L) = \frac{\sum _{k_i \le k_L}^{} \delta \nu _i}{\sum _{i}^{}\delta \nu _i}. \end{aligned}$$

*g*(*k*) is evaluated at increasing limiting absorption coefficient values throughout the full range of absorption coefficients: $k_{min} \le k_L \le k_{max}$ [13].

**Fig. 8:**
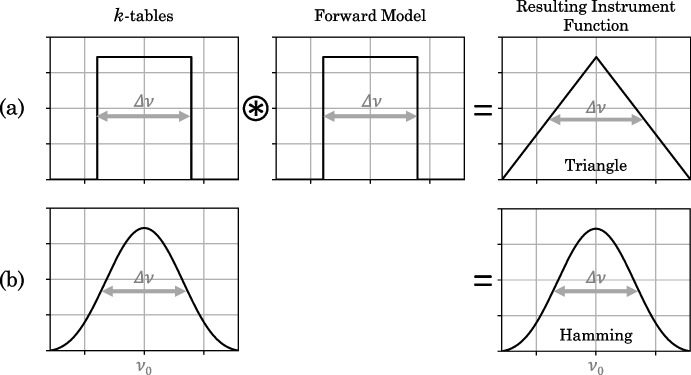
The two instrument functions used in different correlated-*k* (c-*k*) forward models. The first uses *k*-tables that model a square instrument function and the resulting spectrum is convolved with another square function to overall model a triangle instrument function (a). The second uses *k*-tables that model a Hamming instrument function and does not apply a second convolution in the forward model (b). The CIRS apodisation function is a Hamming function [8], that is often approximated as a triangle function for high-SR CIRS observations

Each CFD, *g*(*k*), is then inverted to give a *k*-distribution, *k*(*g*) (e.g., Fig. 7d). The *k*-distribution is sampled at *g*-odinates, $g_i$, with spacings, $\Delta g_i$, distributed with a Gaussian quadrature scheme. This samples the *k*-distribution not at equally spaced ordinates, but rather samples more frequently at the edges of the function. The *k*-distribution is, in general, slowly varying for central values of *g* (e.g., Fig. 7d) and in general has larger gradients at the smallest and lowest values of $g_i$, so a finer $\Delta g_i$ spacing is required at the edges to accurately represent the function. The result is a set of *k*-tables that are suitable for modelling an instrument with a square instrument function of width $\Delta \nu$. In the forward model, the spectrum can be convolved with a second square function of width $\Delta \nu$, such that the resulting instrument function is a triangle function (Fig. 8a). This is considered a reasonable approximation to many instrument line shapes and is the most widely used correlated-*k* approach. For example, it is used in the forward modelling of higher SR CIRS observations (e.g., Teanby et al. [22, 24], Sharkey et al. [20]). The advantage of this standard binning, i.e., using a square function to sample the *k*-distribution, is that a wavenumber shift can be easily added by simply shifting the underlying spectrum. However, low-SR observations require greater attention to forward modelling. For a more accurate forward model, the c-*k* method should include the true instrument function, rather than approximating to a triangle function.

Instead of computing the *k*-distribution with the usual interval CFD, as with equation (11), the *k*-distribution can be computed with a CFD weighted by some apodisation function, $f(\nu )$, such that

12 $$\begin{aligned} g(k_L) = \frac{\sum _{k_i\le k_L}f_i \delta \nu _i}{\sum _{i} f_i \delta \nu _i}, \end{aligned}$$

where $f_i = f(\nu _i - \nu _0)$, and $\nu _0$ is the position of the central peak of the instrument function [12] (Fig. 8). The result is a *k*-distribution for modelling an instrument with a specific apodisation function $f(\nu )$. The instrument function associated with the CIRS instrument is a Hamming function [8] (Fig. 8b), which has apodisation function

13 $$\begin{aligned} f_{Hamm}(\nu ) = \alpha + \beta cos(\frac{\gamma x}{\Delta \nu }), \end{aligned}$$

[3, 27], where $\alpha =$ 0.54, $\beta =$ 0.46, and $\gamma =$ 1.06$\pi$ are constants, and $\Delta \nu =$ 14.25 cm^-1^ is the mean resolution of the CIRS low SR observations. We compute the *k*-distribution using the Hamming function (Equation 13). This produces *k*-tables with the Hamming instrument function directly incorporated, removing the need for a second convolution, and making for a more accurate c-*k* method compared to square *k*-tables (Fig. 8a).

### Using *k*-tables in the forward model

The *k*-distribution of absorption coefficients is pre-tabulated in ‘look-up’ *k*-tables prior to performing a forward model. Modelling an atmosphere as *M* homogeneous plane-parallel layers, each with some constant pressure and temperature $(p_j, T_j)$, and assuming the c-*k* approximation holds, the mean transmission observed at the top of an atmosphere can be evaluated by equation (10). In the forward model, the *k*-distribution, $k_j(g)$, for a single atmospheric layer, *j*, is computed by interpolating the *k*-tables at each atmospheric pressure and temperature $(p_j, T_j)$. If multiple gases contribute to a single absorption, we assume that, in the spectral interval of the bin, the position of the lines of one gas are not correlated with the position of the lines of other gases. The *k*-distributions of *L* gases can then be combined, weighted by the VMR, or mole fraction, $q_l$, of each gas, *l*:

14 $$\begin{aligned} \overline{\mathcal {T}}^{M\,layers,\,L\,gases} = \sum ^{NG}_{i=1} \exp \left[ -\sum _{j=1}^{M}n_{j} \sum _{l}^{L}q_l k_{ijl}(g)\right] \Delta g_i. \end{aligned}$$

The main error in the c-*k* method is here, in the overlapping line approximation.

**Fig. 9:**
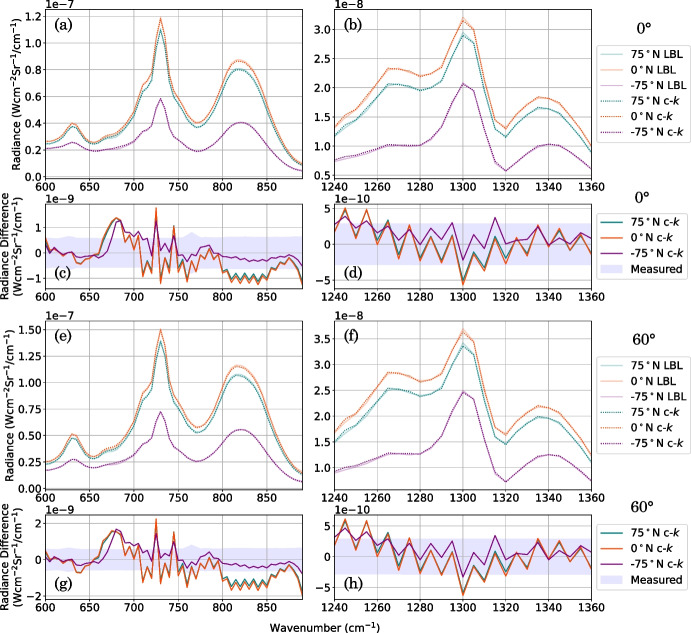
Comparison of the correlated-*k* (c-*k*) to the line-by-line (LBL) forward model. a, b: Synthetic CIRS FP3 (a) and FP4 (b) Titan spectra produced using a LBL forward model with a fine underlying spectral grid spacing ($gs = 2 \times 10^{-4}$ cm^-1^) (solid line), and using a c-*k* forward model with *k*-tables produced with a Hamming instrument (dotted line). Each is performed at three latitude end members: 75^∘^N (blue), 0^∘^N (orange), -75^∘^N (purple) in Titan northern mid-winter (year 2005), assuming a 0^∘^ emission angle. Representative temperature profiles are taken from Teanby et al. [25] (Supplementary Material S3). c, d: LBL-modelled spectra subtracted from c-*k*-modelled spectra. A typical measurement error is shown for comparison (blue shaded region). e–h: The same comparison as in a–d but at an emission angle of 60^∘^. Spectra produced using a c-*k* forward model with Hamming *k*-tables are mostly consistent with the LBL spectra within the measured error

## Accuracy of the correlated-*k* model

Although LBL is the most accurate forward modelling method, the c-*k* method [16] is significantly quicker if performing multiple forward models since the main computation cost is in making *k*-tables, which can be re-used. It also has a potentially small reduction in accuracy compared to the LBL method, if the c-*k* assumptions hold. The critical point here is if the c-*k* is sufficient to model the CIRS spectra to an accuracy better than the measurement noise. To determine this, we compute synthetic CIRS FP3/4 nadir Titan spectra using the c-*k* forward model and compare it to the *gold-standard* LBL-modelled synthetic spectrum convolved with the CIRS instrument function (Hamming function). We perform this at three latitude end members: $75^\circ$N, $0^\circ$N, and $-75^\circ$N (Fig. 9). Typically, nadir observations are used to probe up to a zenith emission angle of around 60^∘^. We perform the comparison of the LBL and c-*k* forward models at two emission angles: 0^∘^ (Fig. 9a–d) and 60^∘^ (Fig. 9e–h), to represent end members.

We compute synthetic CIRS FP3/4 Titan spectra using the c-*k* forward model which uses *k*-tables produced with a Hamming instrument function incorporated directly into the *k*-tables, and omit the second convolution (as outlined in Section 3.1) (Fig. 8b). The *k*-distribution is sampled at *NG* = 50 *g*-ordinates. The spectrum computed using the c-*k* forward model (Fig. 9, dotted lines) is determined to be mostly consistent with the *gold-standard* LBL spectrum (Fig. 9, dashed lines) compared to typically measured errors. The radiance difference between the c-*k* spectrum and the *gold-standard* spectrum is largely within a typical CIRS FP3/4 measurement error (Fig. 9). Hence, we find that the c-*k* method, using *k*-tables produced with the Hamming instrument function, can accurately forward model low-SR CIRS FP3/4 nadir observations of Titan. This is important because many gas features are quite subtle so an inaccurate forward model can introduce significant systematic retrieval artefacts. A c-*k* forward model typically requires $\sim 30$ seconds for FP3 and $\sim 10$ seconds for FP4, wheras we find the LBL requires an average of 115 minutes for FP3 and 36 minutes for FP4 at the optimal grid spacing determined in Section 2.2 (*gs* = $9 \times 10^{-4}$ cm^-1^ for FP3, *gs* = $10 \times 10^{-4}$ cm^-1^ for FP4). The radiance difference quantifies the forward modelling error due to the c-*k* approximation. In an atmospheric retrieval, the forward modelling error influences how much an atmospheric state should be modified in each iteration of the forward model. So a more accurate forward modelling error makes for a more realistic atmospheric retrieval. This error is often taken as a somewhat arbitrary value, constant across the full fitted spectral range. We can now calculate the true forward modelling error for three latitude end members as a function of wavenumber for each focal plane (Appendix A). We determine the root-mean-squared (RMS) forward modelling error to be 0.623 (0^∘^ emission angle), 0.822 (60^∘^ emission angle) nW cm$^{-2}\,$sr$^{-1}\,$/ cm^-1^ for FP3, and 0.231 (0^∘^ emission angle), 0.265 (0^∘^ emission angle) nW cm$^{-2}\,$sr$^{-1}\,$/ cm^-1^ for FP4. These uncertainties are smaller than the typical measurement noise on a CIRS Titan spectrum, which is $\sim 2.5$ nW cm$^{-2}\,$sr$^{-1}\,$/ cm^-1^ for FP3 and $\sim 0.5$ nW cm$^{-2}\,$sr$^{-1}\,$/ cm^-1^ for FP4. These forward modelling errors for the full FP3 and FP4 wavenumber range (Fig. 9) can be used to improve the accuracy of future retrievals of low-SR CIRS FP3/4 nadir observations of Titan.

### Recommendations for practice

We note that some parameters must be constrained to ensure the accuracy of the c-*k* method at forward modelling the low-SR CIRS FP3/4 nadir Titan dataset. Firstly, the underlying spectrum, $k(\nu )$, used to generate the *k*-distributions, *k*(*g*), must be sampled at a sufficient number of points. For example, in NEMESIS, this number of points is evaluated as a function of pressure since fewer points are required at higher pressures. The width of a Hamming function with FWHM $= 14.25$ cm^-1^ including the wings is $\sim 31.4$ cm^-1^. Since we find the optimal grid spacings for LBL forward modelling to be *gs* = $9 \times 10^{-4}$ cm^-1^ (FP3) and *gs* = $10 \times 10^{-4}$ cm^-1^ (FP4), the required number of points should be $\sim (31.4$ cm$^{-1}) / (10 \times 10^{-4}$ cm$^{-1}) \sim 32000$ points. The maximum number of points sampling the underlying spectrum is set to be 32000, which is found to be sufficient in this case.

**Fig. 10:**
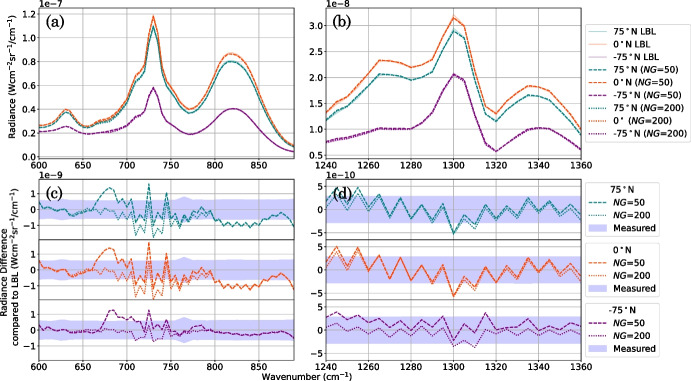
Comparison of synthetic Titan spectra forward modelled using a correlated-*k* (c-*k*) method with Hamming *k*-tables sampled at *NG* = 50 and *NG* = 200 ordinates in cumulative-frequency-space (*g*-space). a, b: Synthetic CIRS FP3/4 Titan spectra produced using a line-by-line (LBL) and a c-*k* forward model. c, d: Radiance difference between the c-*k* and LBL-forward modelled spectra. Shown at three latitude end members: 75^∘^N (blue), 0^∘^N (orange), -75^∘^N (purple) in Titan northern mid-winter (year 2005). A typical CIRS measurement error is shown (c, d, blue shaded region). A c-*k* forward model typically requires $\sim 30$ seconds (FP3) and $\sim 10$ seconds (FP4) for *NG* = 50 and $\sim 140$ seconds (FP3) and $\sim 40$ seconds (FP4) for *NG* = 200, wheras we find the LBL requires an average of 115 minutes (FP3) and 36 minutes (FP4) at optimal grid spacing

The number of *g*-ordiantes, *NG*, at which a *k*-distribution is sampled (Equation 10, Fig. 7d) determines the accuracy and efficiency of a c-*k* forward model. *k*-distributions were sampled using *NG* = 50 (e.g., Fig. 7d) and *NG* = 200 *g*-ordinates (Fig. 10). By equation (7), increasing the number of *g*-ordinates increases the sampling rate and thus allows for a more accurate representation of sharp features near the ends of the *k*(*g*) distribution. Figure 10 shows that increasing the number of *g*-ordinates from *NG* = 50 to *NG* = 200 mostly does not improve the synthetic Titan spectrum significantly, except in the wavenumber region 670–700 cm^-1^: a region where no key gas spectral lines lie (Fig. 2). When the *k*-distribution is sampled at *NG* = 200 *g*-ordinates, the c-*k* forward model has a typical computation run-time of 140 s for FP3 and 40 s for FP4, compared to 30 s for FP3 and 10 s for FP4 when sampled at *NG* = 50. Hence, both the difference in accuracy and computation speed of sampling the *k*-distribution at $NG =$ 50 *g*-ordinates compared to $NG =$ 200 is insignificant compared to the estimated overall forward modelling error (Appendix A). Therefore, we suggest that *NG* = 50 *g*-ordinates are used to model CIRS FP3/4 nadir observations of Titan.

### Synthetic retrieval test

**Fig. 11:**
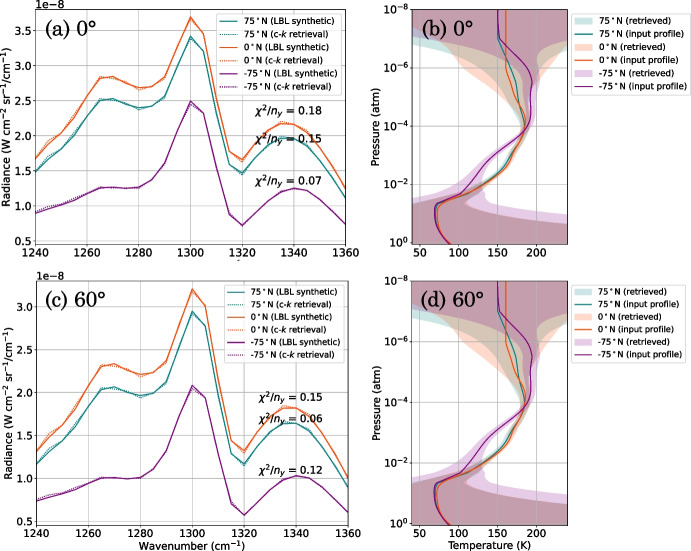
Synthetic retrieval test of the correlated-*k* (c-*k*) forward model. a: Synthetic FP4 Titan spectra were produced using a *gold-standard* ($gs = 2 \times 10^{-4}$ cm^-1^) LBL forward model (solid lines), and a Gaussian noise was added at the minimum FP4 noise level. The FP4 spectra were fitted using a c-*k* forward model with Hamming *k*-tables (dotted lines), to retrieve atmospheric temperature (b). b: The retrieved temperature profile error envelopes (shaded regions) are consistent with the input temperature profiles (solid lines). The test was performed at three latitude end members: 75^∘^N (blue), 0^∘^N (orange), and -75^∘^N (purple) in 2005, assuming a 0^∘^ emission angle. c and d show the same as a and b, but assuming an emission angle of 60^∘^

We test the accuracy of the c-*k* forward model in an atmospheric retrieval by carrying out a synthetic retrieval test. We do this at three example latitude end members: 75^∘^N, 0^∘^N, and -75^∘^N in 2005 to represent northern (mid-winter), equatorial, and southern (early summer) Titan atmospheric states respectively. We first perform a LBL forward model at the *gold-standard* grid spacing ($gs = 2 \times 10^{-4}$ cm^-1^, determined in Section 2.2), to produce a synthetic low-SR Titan FP4 spectrum. Gaussian noise is then added to the spectrum at the minimum CIRS FP4 low-SR spectrum noise level: $NESR/\sqrt{4000}$, with $NESR = 0.5$ nW cm$^{-2}\,$sr^-1^/cm^-1^ [8] (Fig. 11a). We then perform an atmospheric temperature retrieval with the c-*k* forward model, using NEMESIS (Fig. 11a). We find that the retrieved temperature profile is within the error of the input temperature profile used to produce the synthetic spectrum (Fig. 11b). Thus, the retrieval using the c-*k* forward model reliably reproduces the input state.

### Example atmospheric retrieval

**Fig. 12:**
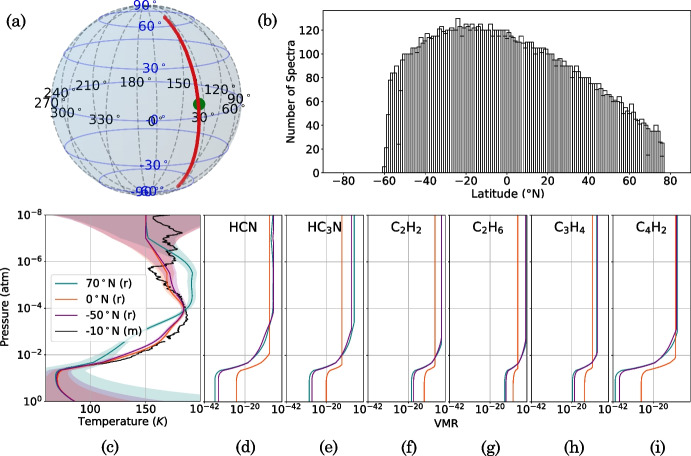
Example Titan temperature and gas volume mixing ratio (VMR) profiles during Titan northern mid-winter (2005). a: Spatial coverage of the CIRS FP3 and FP4 focal planes during an example Titan observation (CIRS$\_013$TI$\_$FIRNADMAP$002\_$PRIME) on a Titan sphere. The centre of each 2^∘^ latitude bin is plotted (red dots). Lines of constant latitude (blue, solid) and longitude (blue, dashed) are shown. The position of the sub-spacecraft point is indicated with a green dot. This observation was acquired at a spectral resolution of FWHM = 14.45 cm^-1^. b: Number of spectra averaged over in each latitude bin, shown for FP3 as an example. Latitude bins have a width of 2^∘^ and are spaced by 1^∘^. c: Example retrieved (‘r’) vertical temperature profile [25] (solid line) and retrieved error (shaded area) at three latitude end members: 50^∘^N (blue), 0^∘^N (orange), and -50^∘^N (purple). The temperature profile measured (‘m’) by the Huygens Atmospheric Structure Instrument (HASI) at approximately -10^∘^N is also shown (black). d–i: VMR vertical profiles for some trace gases in Titan’s atmosphere at the same three latitudes in 2005. Gas vertical profiles are estimated by first assuming a VMR uniform with pressure then applying a condensation level, based on the retrieved temperature profile at that time and latitude. We assume that the VMR does not increase again at higher altitudes as the temperature increases

**Fig. 13:**
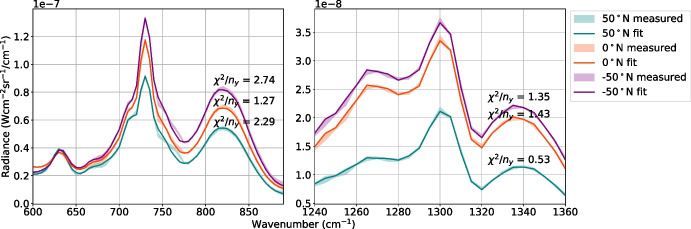
Fits to low-spectral-resolution (SR) CIRS measured Titan spectra (observation CIRS$\_013$TI$\_$FIRNADMAP$002\_$PRIME acquired at FWHM = 14.45 cm^-1^ on 22/08/2005). The retrieval was performed using a correlated-*k* (c-*k*) forward model with *k*-tables produced with the Hamming instrument function. Fitted (solid line) and measured (shaded region) FP3 (a) and FP4 (b) spectra are shown at three example latitudes: 50^∘^N (blue), 0^∘^N (orange) and -50^∘^N (purple). The sub-spacecraft point is within 10^∘^ of the equator during this observation. We use our estimated maximum average forward modelling errors, given in Appendix A. The goodness of fit, $\chi ^2/n_y$, of each spectrum is labelled

Our results suggest low-SR CIRS FP3/4 nadir observations of Titan can be accurately modelled, mostly within measurement error, by using a c-*k* forward model method using *k*-tables produced with a Hamming instrument function (Fig. 9). Here, we assess the effectiveness of the c-*k* forward model in atmospheric retrievals. We fit Titan spectra acquired at low-SR (FWHM = 14.45 cm^-1^) by CIRS FP3/4 on 22/08/2005 (observation CIRS$\_013$TI$\_$FIRNADMAP$002\_$PRIME). This observation scanned with a nadir viewing geometry at an approximately constant longitude (Fig. 12) and is binned in 2^∘^ latitude bins. We use NEMESIS [12] to perform a 2-stage retrieval similar to that of Teanby et al. [25], where, first, temperature is retrieved from the CH_4_–$\nu _4$ band, then key gases are retrieved from FP3 with a fixed temperature profile. We use the c-*k* forward model, specifying the wavenumber-dependent forward modelling uncertainty determined in Section 4. Figure 13 shows the retrieved FP3/4 spectrum at three latitude end members: 50^∘^N, 0^∘^N, and -50^∘^N representing northern (mid-winter), equatorial, and southern (early summer) atmospheric states respectively. Since the sub-spacecraft point is approximately stationary at the equator during this observation, emission angles of these three end members are approximately 50^∘^ for ±50^∘^N, and approximately 0^∘^ for 0^∘^N. The fitted spectra are generally within the measurement error, except in the region 740 - 775 cm^-1^, where propane (C_3_H_8_) is not well resolved. However, this region is also not well resolved in the LBL retrievals with the *gold-standard* spectral grid spacing (*gs* = $2 \times 10^{-4}$ cm^-1^), so this inaccuracy is unlikely a result of the forward model. The goodness of fit is $\chi ^2/n_y =$ 2.29 ($50^{\circ }$N), 1.27 ($0^{\circ }$N), 2.74 ($-50^{\circ }$N) for FP3, and 0.53 ($50^{\circ }$N), 1.43 ($0^{\circ }$N), 1.35 ($-50^{\circ }$N) for FP4. The goodness of fit is close to 1 for all three latitude end members, but is best at the equator for both FP3 and FP4. This is expected as spectral lines are generally wider at smaller emission angles which probe deeper in the atmosphere. Observations at a lower emission angle have a shorter path length through the atmosphere, and hence probe a lower altitude, where atmospheric pressure is higher.

## Conclusion

Cassini/CIRS low spectral resolution (SR) (FWHM = 12.6–14.7 cm^-1^) observations of Titan comprise more than a third of all CIRS Titan observations, and typically provide the best spatial resolution and coverage for observing Titan’s complex atmosphere. However, low-SR observations have subtle and blended peaks, so greater attention is required to accurately forward model this vast dataset using the correlated-*k* approximation. We assess the accuracy of the computationally-efficient c-*k* forward model and show that the line-by-line (LBL) forward model, despite its accuracy, is typically computationally inefficient. We provide solutions to this.

- LBL forward modelling requires Nyquist line sampling at a grid spacing less than the width of the narrowest spectral line, but we show this can be relaxed for CIRS FP3/4 low-SR nadir observations depending on noise requirements. The LBL method may be advantageous if performing a small number of forward models, as it avoids having to make *k*-tables, which can require a one-time large computation cost.
- We show that CIRS FP3/4 low-SR nadir observations can be accurately and efficiently modelled using a c-*k* forward model with a *k*-distribution sampled using the CIRS instrument function (Hamming function) at 50 ordinates in cumulative-frequency-space.
- We estimate a wavenumber-dependent forward-modelling error (Fig. 9), which can be used to improve the rigour of future atmospheric retrievals of low-SR Cassini CIRS FP3/4 nadir observations of Titan.

These results will allow accurate and efficient analysis of this large CIRS spectral dataset. More generally, we recommend extra care should be taken when modelling spectral observations with a low SR compared to the width of the narrowest spectral line in the region of interest. We suggest comparing the c-*k* to the LBL forward model to determine forward modelling errors that can be used to improve the accruacy of retrievals. We have shown that computationally-efficient and accurate forward modelling can be achievable, but that extra care should be taken when modelling such observations.

## Data Availability

The Cassini CIRS data are available from NASA’s Planetary Data System (https://pds.nasa.gov).

## Appendix A: Forward modelling errors

**Table 2:** Estimated uncertainty in forward modelling low spectral resolution (FWHM $\sim 14.5$ cm^-1^) Cassini CIRS FP3/4 nadir observations of Titan using a correlated-*k* (c-*k*) forward model

Wavenumber (cm^-1^)	Forward Modelling Error (Wcm^-2^sr$^{-1}/$cm^-1^)		
	0^∘^	60^∘^	Maximum
FP3			
600.0	4.73e-10	3.57e-10	3.57e-10
605.0	5.22e-11	5.59e-11	5.59e-11
610.0	1.67e-10	1.07e-10	1.07e-10
615.0	3.70e-11	4.58e-11	4.58e-11
620.0	1.05e-10	1.70e-10	1.70e-10
625.0	3.37e-12	2.76e-11	2.76e-11
630.0	2.39e-11	4.10e-11	4.10e-11
635.0	3.11e-10	5.58e-10	5.58e-10
640.0	3.48e-10	5.89e-10	5.89e-10
645.0	2.14e-10	3.06e-10	3.06e-10
650.0	1.85e-10	2.48e-10	2.48e-10
655.0	8.47e-11	4.79e-11	4.79e-11
660.0	1.08e-10	1.67e-10	1.67e-10
665.0	3.64e-10	6.53e-10	6.53e-10
670.0	6.11e-10	9.57e-10	9.57e-10
675.0	9.97e-10	1.39e-09	1.39e-09
680.0	1.32e-09	1.59e-09	1.59e-09
685.0	1.27e-09	1.43e-09	1.43e-09
690.0	5.44e-10	4.58e-10	4.58e-10
695.0	7.32e-10	8.07e-10	8.07e-10
700.0	2.92e-10	2.91e-10	2.91e-10
705.0	7.16e-10	8.20e-10	8.20e-10
710.0	7.46e-10	9.34e-10	9.34e-10
715.0	4.24e-10	4.30e-10	4.30e-10
720.0	5.47e-10	6.87e-10	6.87e-10
725.0	1.51e-09	1.90e-09	1.90e-09
730.0	7.41e-10	8.45e-10	8.45e-10
735.0	2.14e-10	2.37e-10	2.37e-10
740.0	4.60e-10	6.69e-10	6.69e-10
745.0	9.27e-10	1.34e-09	1.34e-09
750.0	4.93e-10	5.41e-10	5.41e-10
755.0	2.26e-10	1.86e-10	1.86e-10
760.0	8.41e-10	1.12e-09	1.12e-09
765.0	2.26e-10	2.93e-10	2.93e-10
770.0	5.32e-10	7.95e-10	7.95e-10
775.0	1.27e-10	1.85e-10	1.85e-10
780.0	2.44e-10	4.02e-10	4.02e-10
785.0	2.04e-10	2.22e-10	2.22e-10
790.0	1.83e-10	3.44e-10	3.44e-10
795.0	4.45e-11	5.84e-11	5.84e-11
800.0	5.32e-10	8.17e-10	8.17e-10
805.0	4.78e-10	7.40e-10	7.40e-10
810.0	7.94e-10	1.15e-09	1.15e-09
815.0	6.45e-10	8.51e-10	8.51e-10
820.0	8.63e-10	1.18e-09	1.18e-09
825.0	6.17e-10	8.57e-10	8.57e-10
830.0	8.41e-10	1.18e-09	1.18e-09
835.0	6.47e-10	8.70e-10	8.70e-10
840.0	8.81e-10	1.22e-09	1.22e-09
845.0	6.58e-10	8.94e-10	8.94e-10
850.0	8.00e-10	1.14e-09	1.14e-09
855.0	4.58e-10	6.39e-10	6.39e-10
860.0	5.05e-10	7.42e-10	7.42e-10
865.0	2.89e-10	4.05e-10	4.05e-10
870.0	3.53e-10	5.10e-10	5.10e-10
875.0	1.47e-10	2.20e-10	2.20e-10
880.0	3.05e-10	4.57e-10	4.57e-10
885.0	5.29e-10	8.04e-10	8.04e-10
890.0	9.65e-10	1.51e-09	1.51e-09
FP4			
1240.0	2.05e-10	2.28e-10	2.28e-10
1245.0	4.55e-10	5.53e-10	5.53e-10
1250.0	1.38e-10	1.68e-10	1.68e-10
1255.0	4.33e-10	5.21e-10	5.21e-10
1260.0	7.47e-11	8.23e-11	8.23e-11
1265.0	3.03e-10	3.51e-10	3.51e-10
1270.0	1.37e-10	1.33e-10	1.33e-10
1275.0	2.43e-10	2.76e-10	2.76e-10
1280.0	1.53e-10	1.87e-10	1.87e-10
1285.0	1.45e-10	1.41e-10	1.41e-10
1290.0	1.87e-10	2.00e-10	2.00e-10
1295.0	1.77e-10	1.36e-10	1.36e-10
1300.0	4.29e-10	5.19e-10	5.19e-10
1305.0	1.34e-10	1.23e-10	1.23e-10
1310.0	2.57e-10	3.10e-10	3.10e-10
1315.0	1.85e-10	1.62e-10	1.62e-10
1320.0	1.61e-10	2.04e-10	2.04e-10
1325.0	7.17e-11	6.33e-11	6.33e-11
1330.0	1.24e-10	1.38e-10	1.38e-10
1335.0	2.62e-10	2.68e-10	2.68e-10
1340.0	3.1e-11	4.28e-11	4.28e-11
1345.0	1.72e-10	1.98e-10	1.98e-10
1350.0	5.28e-11	5.51e-11	5.51e-11
1355.0	1.4e-10	1.55e-10	1.55e-10
1360.0	1.19e-10	1.55e-10	1.55e-10

Uncertainties are taken as the difference between synthetic spectra forward modelled using a *gold-standard* line-by-line (LBL) method and a c-*k* method. The values shown are the average over three sets of synthetic spectra, produced using representative temperature profiles from Teanby et al. [25] (Supplementary Material S3) at latitude end-members 75^∘^N, 0^∘^N, and -75^∘^N in Titan northern mid-winter (year 2005). Average forward modelling errors were calculated at two emission angles: 0^∘^ and 60^∘^. The *k*-tables used in the c-*k* forward model are produced using a Hamming instrument function and sampled at *NG* = 50 *g*-ordinates.. Uncertainties can be used in future forward modelling of this dataset

## Code availability

The radiative transfer code is fully described in Irwin et al. [12].
